# Restrictive Expression of Acid-Sensing Ion Channel 5 (Asic5) in Unipolar Brush Cells of the Vestibulocerebellum

**DOI:** 10.1371/journal.pone.0091326

**Published:** 2014-03-24

**Authors:** Nina Boiko, Volodymyr Kucher, Bin Wang, James D. Stockand

**Affiliations:** Department of Physiology, University of Texas Health Science Center, San Antonio, Texas, United States of America; The Ohio State University, United States of America

## Abstract

Acid-sensing ion channels (Asic) are ligand-gated ion channels in the Degenerin/Epithelial Na^+^ channel (Deg/ENaC) family. Asic proteins are richly expressed in mammalian neurons. Mammals express five Asic genes: *Asic1-5*. The gene product of *Asic5* is an orphan member of the family about which little is known. To investigate Asic5 expression, we created an *Asic5* reporter mouse. We find that *Asic5* is chiefly expressed in the brain in the cerebellum, specifically in the ventral uvula and nodulus of the vestibulocerebellum. Here, *Asic5* is restrictively expressed in a subset of interneurons in the granular layer. The locale, distinctive shape and immunohistochemical properties of these *Asic5*-expressing interneurons identify them as unipolar brush cells (UBC). *Asic5* is richly expressed in a subset of UBCs that also express the metabotropic glutamate receptor 1α (mGluR1α) but not those that express calretinin. Results from single cell RT-PCR and electrophysiological examination of these cells are consistent with this identity. Such observations are consistent with Asic5 playing a key role in the physiology of UBCs and in the function of the vestibulocerebellum.

## Introduction

Degenerin/Epithelial Na^+^ channel (Deg/ENaC) proteins comprise an ancient ion channel family that is expressed in every metazoan species [1]. Mammals express two general types of Deg/ENaC channels: acid-sensing ion channels (Asic) and ENaC [2]–[5]. ENaC is chiefly expressed in epithelial cells and is involved in Na^+^ transport. Asics are expressed in neurons of the peripheral and central nervous systems. Asics are ligand-gated channels that conduct depolarizing inward Na^+^ currents. This allows certain Asics, as typified by Asic1 in hippocampal CA1 neurons, to function as post synaptic ionotropic receptors important to synaptic transmission [6]. Because they are sensitive to acid, Asic proteins also contribute to excitotoxic neuronal damage during ischemia and local acidosis [3], [7], [8]. Inappropriate Asic activity contributes to the progression and symptoms of several pathologies of the brain to include stroke and multiple sclerosis [8]–[11]. Consequently, these channels are an emerging target to treat such diseases. Sensitivity to acid also underlies involvement of Asics in pain sensing and them being targets for analgesics [8], [12].

Mammals express five Asic genes: *Asic1-5*. While relatively much is understood about Asic1-3 and to a lesser extent Asic4, Asic5 is an orphan member of the family about which little is known. Asic5 has been called the brain, liver, intestinal Na^+^ channel (BLINaC), human intestinal Na^+^ channel (hINaC), and bile acid-sensitive ion channel (BASIC) previously [13]–[15]. Asic5 mRNA is richly expressed in the brain and liver [13], [14]. In the brain, Asic5 mRNA is most abundant in the cerebellum and sparse in other areas [13]. In the liver, Asic5 is expressed in the luminal membrane of epithelial cholangiocytes that line the bile duct and is activated by bile acid [15], [16]. This is consistent with Asic5 serving a Na^+^ transport function akin to that of ENaC. The function of Asic5 in the brain remains obscure. Moreover, it is unknown if Asic5 is expressed in neurons or other brain cell types.

The goal of this study was to identify the specific region and type of cell in the brain that expresses *Asic5* to begin understanding the function of this orphan Deg/ENaC channel in the nervous system. Findings from a novel *Asic5* reporter mouse combined with single cell RT-PCR and electrophysiology demonstrate that *Asic5* is restrictively expressed in interneurons in the granular layer of the cerebellum, specifically in unipolar brush cells (UBC) of the vestibulocerebellum.

## Materials and Methods

### Materials

All chemicals and materials were purchased from Sigma unless noted otherwise. The Tg(Grp-EGFP)DV197Gsat mouse used in these studies was generated by the GENSAT project [17]–[19] and was obtained with permission from Drs. M. Martina and E. Mugnaini (Northwestern University, Evanston IL). In this transgenic animal, GFP is primarily expressed in a subset of vestibulocerebellar UBCs that also express mGluR1α.

### Animal use and care

All animal use and welfare adhered to the National Institutes of Health Guide for the Care and Use of Laboratory Animals following a protocol reviewed and approved by the Institutional Animal Care and Use Committee of the University of Texas Health Science Center at San Antonio. Experimental mice were housed and cared for by Laboratory Animal Resources at the University of Texas Health Science Center at San Antonio, which is fully accredited by the Association for Assessment and Accreditation of Laboratory Animal Care, and licensed by the United States Department of Agriculture.

### Generation and genotyping of the Asic5 reporter mouse

The *Asci5^tm2a(KOMP)Wtsi^* targeting vector (ID: 33140) was purchased from the Knockout Mouse Project (KOMP) Repository at the University of California Davis and Children's Hospital Oakland Research Institute. This targeting vector contains the SA-βgeo-pA gene trapping cassette and produces a knockout first allele that is reporter-tagged and has conditional potential. This targeting vector contains a splice acceptor (En2 SA) and a polyadenylation tract (pA) upstream and downstream, respectively, of *lacZ*, and has a downstream *neo* gene driven by a separate promoter. Exon2 and 3 encode nucleotides 41–348 and 349–587 of the *Asic5* coding sequence (Transcript ID: ENSMUST00000029641). The intron between exon2 and 3 is 4555 nucleotides long. The SA-βgeo-pA trapping cassette replaces one hundred nucleotides of this intronic sequence in the *Asic5^tm2a(KOMP)Wtsi^* targeting vector.

Congenic germline transmitting chimeras were generated for the Stockand laboratory by the Mouse Biology Program at the University of California Davis through a fee for service agreement. In brief, the *Asic5^tm2a(KOMP)Wtsi^* targeting vector was electroporated into JM8A3.N1 (Agouti) embryonic stem cells [20]. A suitable clone positive for homologous recombination was then injected into blastocysts from a B6D2F1×C57BL/6 donor.

Mouse genomic DNA was extracted from tail samples using the DNeasy Blood and Tissue Kit (Qiagen) following the manufacture's instructions. For genotyping, a standard polymerase chain reaction was performed with this genomic DNA using the MangoMix PCR Kit (Bioline). The allele specific primers used in these reactions were: 1. forward common 5′-AGCTGACTGCTGGCTGGTTATGTG-3′; 2. reverse wild type 5′-TTTGGCTTAACTGACCATAAAGGC-3′; and 3. reverse reporter 5′-TCCTCCTACATAGTTGGCAGTGTTT-3′.

Long range PCR was used to confirm homologous recombination and to define the site of insertion. For long range PCR, genomic DNA was isolated as above and amplified using Phusion Hot Start II High-Fidelity DNA Polymerase (Fisher Scientific). The forward 5′-AGCCTCTGATTGCAGATGGACCTCA-3′ primer, which is outside the homology arm, was used in combination with the reverse 5′-GGTGGTGTGGGAAAGGGTTCGAAG-3′ primer, which is unique to the targeting vector, to amplify across the 5′ insertion site. Following homologous recombination, this primer set will produce a 5,342 bp product with no product amplified from the wild type allele. The forward 5′-AGCCTCTGATTGCAGATGGACCTCA-3′ primer, which is unique to the targeting vector, was used in combination with the reverse 5′-GGTGGTGTGGGAAAGGGTTCGAAG-3′ primer, which is outside the homology arm, to amplify across the 3′ insertion site. Following homologous recombination, this primer set will produce a 5,110 bp product with no product amplified from the wild type allele.

### RT-PCR

RNA was isolated from the cerebellum and liver using the RNeasy Kit (Qiagen) according to the manufacturer's instructions. First strand cDNA was prepared using the ProtoScript First Strand cDNA Synthesis Kit (NE BioLabs). PCR was then performed using the iProof High Fidelity PCR Kit (Bio-Rad Laboratories). The primers used to identify lacZ and β-actin mRNA were: forward 5′-GATTACCGTTGATGTTGAAGTGG-3′ and reverse 5′-GGGAAGACGTACGGGGTATACAT-3; and forward 5′-TTCCTTCTTGGGTATGGAATCCTG-3′ and reverse 5′- GTGTAAAACGCAGCTCAGTAACAGT-3′, respectively.

### Single-cell RT-PCR

Single cell RT-PCR followed standard protocols. In brief, the vestibulocerebellums of Tg(Grp-EGFP)DV197Gsat mice were dissected away from meninges in ice-cold Hank's Balanced Salt Solution (HBSS), cut into small pieces and digested with 3 mg/ml protease XXIII at 31°C for 7 minutes [21]–[23]. Digestion was terminated with 1 mg/ml BSA and 1 mg/ml trypsin inhibitor. A dissociated cell suspension was created from this digestion by mechanical trituration. Dissociated neurons were plated on poly-D-lysine-coated glass coverslips in Tyrode's solution. GFP-positive UBCs were identified by morphology and GFP emissions. Individual GFP-positive UBCs and GFP-negative vestibulocerebellar neurons were captured in a glass micropipette filled with Tyrode's solution using gentle suction. RNA from individual cells was isolated and reverse transcribed using the SuperScript III CellsDirect cDNA Synthesis System (Life Technologies). PCR was then performed as above with the iProof High Fidelity PCR Kit (Bio-Rad Laboratories). Primers used to detect Asic5 and actin mRNA in these reactions were: forward 5′- AGTCGTTTGGTCAATTACTTCACAT-3′ and reverse 5′- AAAAACCATTGTTCTTGACAAACTC-3′; and forward 5′-TTCCTTCTTGGGTATGGAATCCTG-3′ and reverse 5′- GTGTAAAACGCAGCTCAGTAACAGT-3′, respectively

### Staining for β-galactosidase activity

Deeply anesthetized mice were perfused transcardially with saline and then ice cold 2% paraformaldehyde in PBS. Sagittal and coronal (200 µm) sections where prepared using a vibratome from the brains or cerebellums of these mice. Sections were stained with β-Gal substrate following a standard protocol [24]. In brief, sections were post-fixed with 0.2% glutaraldehyde, permeabilized with 0.2% Tween 20 and stained overnight at 37°C with 1 mg/ml X-Gal in PBS supplemented with 5 mM K3[Fe(CN)6], 5 mM K4[Fe(CN)6], 2 mM MgCl_2_ and 0.2% Tween 20. Images of β-galactosidase activity in cerebellar sections were acquired using a MZ16 stereomicroscope (Leica).

### Immunohistochemistry

Perfusion fixed murine brains were harvested as above and subsequently fixed an additional two hours at 4°C with 2% paraformaldehyde. Brains were then cryoprotected with 20% glucose at 4°C for 24–48 hours. Sagittal cryosections (14 µm) through the vermis of dissected cerebellums were prepared on a freezing-stage microtome and subsequently mounted on charged superfrost-plus glass slides. Sections were blocked for 30 minutes at room temperature with 3% BSA in PBS containing 0.1% Triton X-100 and then incubated with primary antibody at 4°C overnight. Sections then were exposed to appropriate secondary antibodies as normal. Secondary antibodies were conjugated to Alexa 488 or Alexa 568 (Life Technologies). Fluorescence images of cerebellar sections and UBCs were acquired using an IX81/FV-1000 confocal microscope (Olympus) housed within the Core Optical Imaging Facility at UTHSCSA. The following primary antibodies were used in the current studies: rabbit anti-β-galactosidase (1∶5,000; MP Biomedicals), mouse anti-mGluR1α (1∶200; BD Biosciences), mouse anti-calretinin (1∶1000; Millipore), and mouse anti-GFP (1∶5000; Millipore). Cerebellum sections probed with mouse primary antibodies were treated with M.O.M IgG blocking reagent (Vector Laboratories) to minimize nonspecific cross reactivity.

### Electrophysiology in cerebellar slices

Brain slices were prepared from 3–4 week old mice following proven protocols used previously to record from vestibulocerebellar granular layer interneurons to include UBCs [25], [26]. In brief, freshly harvested cerebellums were placed in ice-cold cutting solution containing (in mM): 87 NaCl, 25 NaHCO_3_, 2.5 KCl, 1.25 NaH_2_PO_4_, 0.5 CaCl_2_, 7 MgCl_2_, 75 sucrose, 25 glucose, 0.4 vitamin C and 1 kynurenic acid, bubbled with 95% O_2_-5% CO_2_. Parasagittal slices (300 µm) were prepared using a vibratome. Slices then were maintained in a physiological extracellular solution containing (in mM) 125 NaCl, 25 NaHCO_3_, 1.25 NaH_2_PO_4_, 2.5 KCl, 1.2 CaCl_2_, 1 MgCl_2_, 25 glucose and 0.4 vitamin C (bubbled with 95%O_2_-5% CO_2_) at 30°C for 1 hour and then moved to room temperature.

For patch-clamping, interneurons in the granular layer of lobules IX and X were visualized with infrared/DIC videomicroscopy using a 60× water-immersion objective on an upright BX51WI microscope (Olympus). GFP-expressing UBCs were identified with epifluorescence illumination. Patch electrodes (5–9 mΩ) were fabricated from borosilicate glass capillaries (B150-86-10, Sutter Instrument) using a horizontal puller. The intracellular recording solution contained (in mM): 140 K-gluconate, 2 MgCl_2_, 10 EGTA, 2 Na_2_ATP, 0.1 NaGTP and 10 Hepes, pH 7.3. Whole-cell current-clamp recordings were performed using a HEKA EPC10 amplifier and the Patchmaster software (HEKA Instruments). Data were sampled at 40 µs intervals and low-pass filtered at 7.2 kHz using a four-pole Bessel filter. Neurons were held at −70 mV. Input resistance was estimated using a −20 pA current injection. Action potentials were elicited by supra-threshold current injections of 1 second.

### Data Analysis and Comparison

Contrast maps of β-galactosidase activity were generated using paint.net with the Selective Enhancer plug-in. Plug-in parameters were set to maximize contrast. Transverse renderings are maximal intensity projections of the 3D-stacks. Sections in these transverse renderings were aligned according to the Allen Brain Atlas (www.brain-map.org).

Analysis of action potentials was performed using the AxoGraphX software (Kagi). For evaluation of individual action potential properties, the 1^st^ and 2^nd^ action potential waveforms elicited by a 30-pA current injection were used. Data were statistically analyzed using KaleidaGraph (Synergy Software) and graphed using Igor Pro (WaveMetrics).

All summarized data reported as mean ± SEM. Unpaired data and proportions were compared with a *t*-test and Z-test, respectively. The criterion for significance was *P*≤0.05.

## Results

### The *Asic5^tm2a(KOMP)Wtsi^* mouse

**Figure 1:**
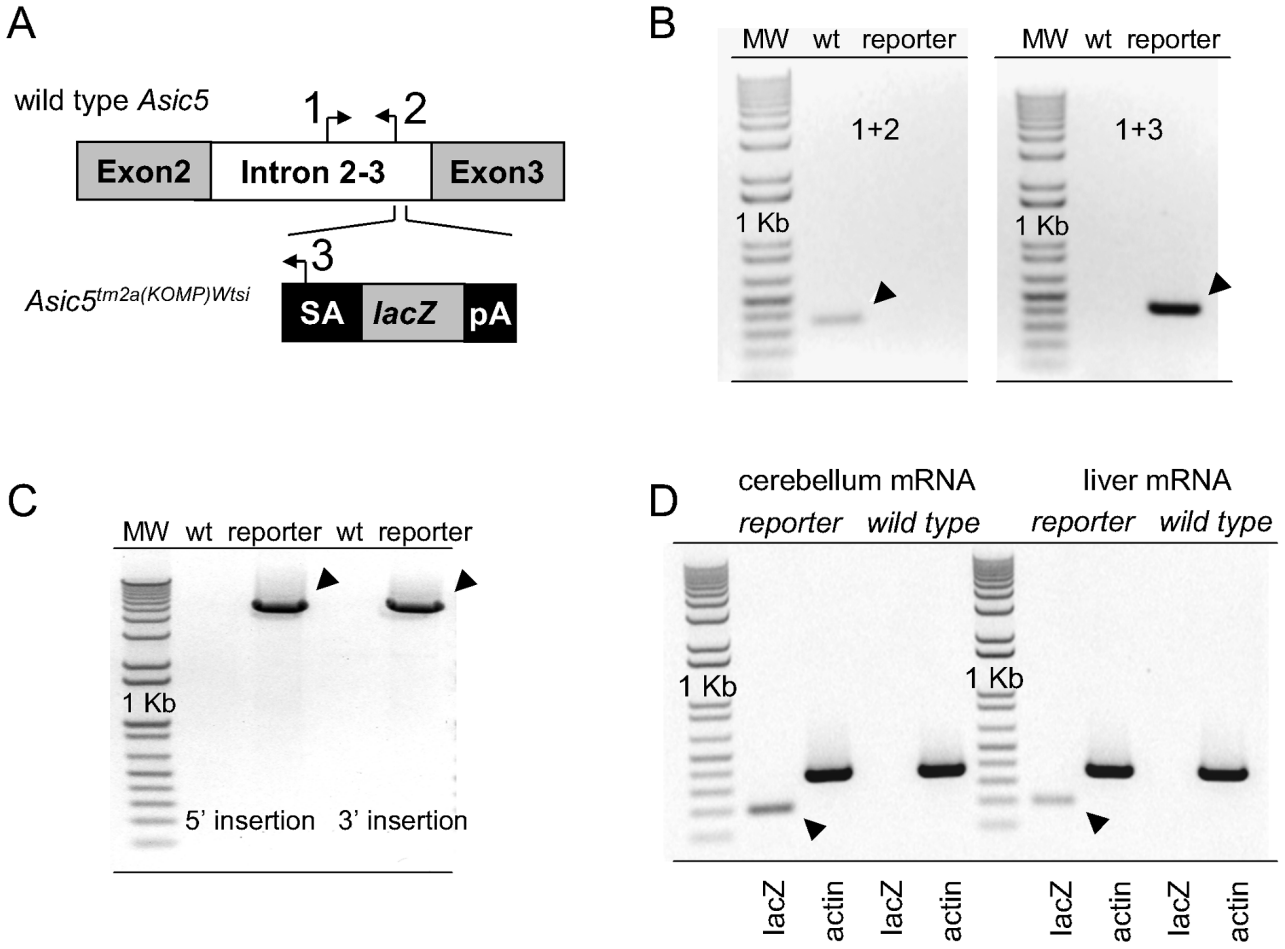
The *Asic5^tm2a(KOMP)Wtsi^* mouse. A. A simplified scheme showing the wild type *Asic5* (top) and reporter *Asic5^tm2a(KOMP)Wtsi^* (bottom) alleles as well as the orientation and relative positions of binding sites for primers used in genotyping reactions. The *neo* gene in the trapping cassette is not shown in this figure. B. Inverted images of representative gels (n≥60) containing typical products from genotyping reactions of homozygous wild type and *Asic5^tm2a(KOMP)Wtsi^* reporter mice using primers 1, 2 & 3. Expected products are indicated with arrowheads. C. Inverted image of a representative gel (n>3) containing the products of long range PCR reactions across both insertion arms. D. Inverted images of representative gels containing typical RT-PCR products generated from mRNA isolated from the cerebellum (left) and liver (right) of *Asic5^tm2a(KOMP)Wtsi^* mice and wild type littermates. Primers specific for lacZ and actin mRNAs were used in these reactions with actin serving as a positive control. Arrowheads indicate the expected lacZ product.

The simplified scheme shown in figure 1A depicts the wild type *Asic5* and *Asic5^tm2a(KOMP)Wtsi^* alleles. Also shown are the relative binding sites and orientation of genotyping primers. The forward common primer 1 interacts with a sequence within intron 2–3 that is in both the wild type and reporter allele. The reverse wild type primer 2 interacts with an intronic sequence in the wild type allele not found in the reporter allele. The reverse reporter primer 3 interacts with sequence unique to the gene trapping cassette and thus is only in the *Asic5^tm2a(KOMP)Wtsi^* allele. Figure 1B shows typical results from genotyping experiments on mice homozygous for the wild type or reporter allele using these primers. As expected the combination of primers 1 and 2 produced a PCR product of predicted size from wild type mice that was absent in *Asic5^tm2a(KOMP)Wtsi^* mice. Primers 1 and 3 produced a product of predicted size from reporter mice that was absent in wild type littermates.

To confirm proper genomic targeting, the site of insertion of the targeting vector was mapped with long range PCR across both insertion arms. As shown in figure 1C insertion was at the expected site.

Figure 1D shows typical results from RT-PCR experiments probing expression of lacZ mRNA in the cerebellum and liver of homozygous *Asic5^tm2a(KOMP)Wtsi^* mice and wild type littermates. Asic5 mRNA has previously been shown to be expressed in these tissues [13], [14]. As expected, lacZ mRNA was expressed in the cerebellums and livers of *Asic5^tm2a(KOMP)Wtsi^* mice but not wild type littermates.

### *Asic5* is restrictively expressed in interneurons in the granular layer of the vestibulocerebellum

**Figure 2:**
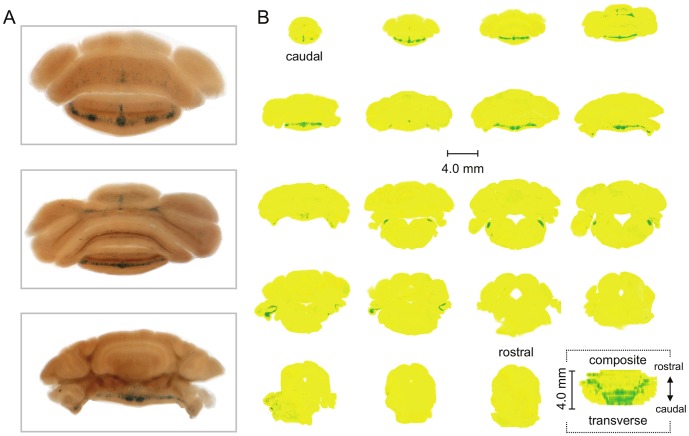
β-Gal is restrictively expressed in the vestibulocerebellum of the *Asic5^tm2a(KOMP)Wtsi^* mouse. A. Representative images of coronal sections (200 µm) through the cerebellum and brainstem of the reporter mouse stained for β-galactosidase activity (blue). Slices are displayed in a caudal to rostral arrangement with the top being the most caudal of the three. The top, middle and bottom slices correspond with the 2^nd^, 4^th^ and 8^th^ sections shown in 2B. B. Nineteen sequential coronal sections through the entire cerebellum (and part of the brainstem) of the *Asic5^tm2a)KOMP)Wtsi^* mouse. Sections are shown in a caudal to rostral arrangement with the top left being the most caudal and the lower right (second to last) being the most rostral. Raw data are identical to that shown in 2A. Images were color and contrast manipulated to emphasize staining for β-galactosidase activity with yellow representing no staining and green representing robust staining. The final image in 2B represents a composite of the whole cerebellum (and part of the brainstem) collapsed into a 2-D rendering shown in the transverse plane. This rendering is a compilation of the 19 coronal sections shown in 2B stacked caudal to rostral, flipped 90° and collapsed.

Figure 2A shows typical staining for β-galactosidase activity in three representative coronal slices of the cerebellum from the *Asic5^tm2a(KOMP)Wtsi^* mouse. Figure 2B shows a contrast map of β-galactosidase staining in sequential sections through the entire cerebellum (and a portion of the brainstem). Raw data were from experiments identical to that in 2A. Also shown in 2B is a composite contrast rendering of the whole cerebellum (and part of the brainstem) viewed from the transverse plane. In the reporter animal, staining of β-galactosidase activity in the cerebellum was strong and chiefly restricted to the ventrocaudal region of the vermis in the posterior and flocculonodular lobes corresponding to the vestibulocerebellum; and in the brainstem in the dorsal cochlear nucleus. Little staining was observed in the brain of the reporter mouse outside of the cerebellum and no staining was seen in the cerebellum of wild type littermates. Staining outside of the cerebellum in the brain of the reporter mouse, as shown in figure S1, was sparse and punctuate in nature being localized to a small region of the cortex and the olfactory tubercle.

**Figure 3:**
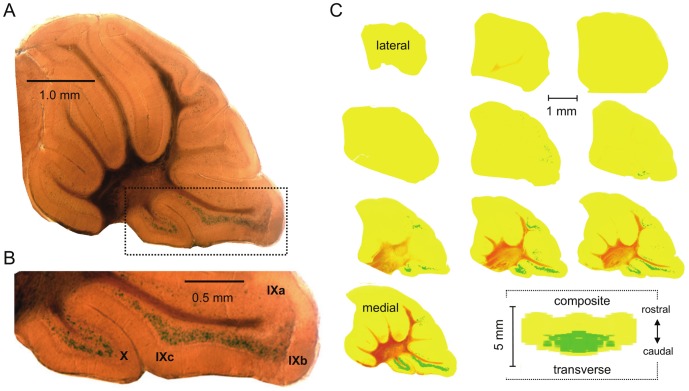
*Asic5* is restrictively expressed in interneurons in the granular layer. A. Representative midsagittal section (200 µm) of the cerebellum from an *Asic5^tm2a(KOMP)Wtsi^* mouse stained for β-galactosidase activity. B. Lobules IX and X in the (boxed) section in 3A shown at a magnified scale. C. Ten sequential sagittal sections through one half of the entire cerebellum of the reporter mouse. Sections are shown in a lateral to medial arrangement with the top left being the most lateral and the lower right (second to last) closest to the midline. Raw data are identical to that shown in 3A. Images were color and contrast manipulated to emphasize staining for β-galactosidase activity with yellow representing no staining and green representing robust staining. The final image in 3C represents a composite of the whole cerebellum collapsed into a 2-D rendering shown in the transverse plane. This rendering is a compilation of the 10 sagittal sections shown in 3B stacked lateral to medial, flipped 90° and collapsed with the left half of this figure being a mirror image of the right.

Shown in figure 3A with an enlargement of the area containing the vestibulocerebellum reproduced in 3B is a representative midsagittal section of the cerebellum of the *Asic5^tm2a(KOMP)Wtsi^* mouse stained for β-galactosidase activity. As above for coronal sections, figure 3C shows a contrast map of β-galactosidase staining in sagittal sections through half of the cerebellum and a composite contrast rendering of staining in the whole cerebellum viewed from the transverse plane. β-galactosidase activity was restricted to interneurons in the granular layer of the vestibulocerebellum chiefly in lobules X and IXb and IXc, but notably not IXa. These regions of the mouse cerebellum correspond to the ventral uvula and nodulus in the human.

### *Asic5* is expressed in unipolar brush cells

**Figure 4:**
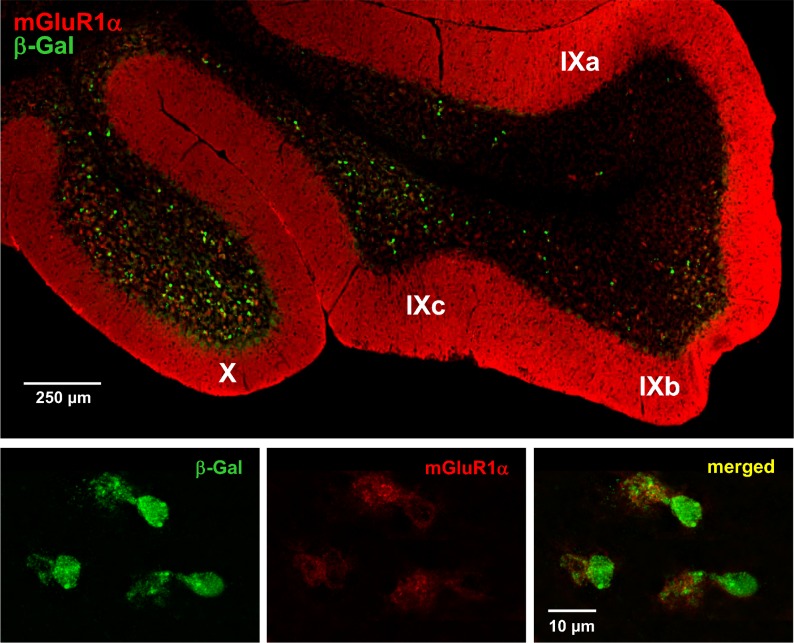
*Asic5* is expressed in mGluR1α (+) UBCs. A representative fluorescence micrograph showing lobules IX and X in a typical midsagittal section of the cerebellum from the *Asic5^tm2a(KOMP)Wtsi^* mouse stained with anti-β-Gal (green) and anti-mGluR1α (red) antibodies. (This experiment was repeated with similar results in more than five different mice.) A region of lobule X that contains representative UBCs positive for both β-Gal (green; left) and mGluR1α (red; middle) is shown at a magnified scale below with a merged image shown to the right.

UBCs are excitatory glutamatergic interneurons common to the granular layer of the vestibulocerebellum, lobules X and IXb-c but not IXa, that are sparse throughout the rest of the cerebellum and brain [27]–[30]. UBCs have a characteristic morphology. As shown in figure 4 and figures S2, S3, S4, β-Gal expressing cerebellar interneurons in the *Asic5^tm2a(KOMP)Wtsi^* mouse had a distinctive morphology including a short but broad dendrite that terminated in an extended dendriole. Such morphology is a hallmark of UBCs and was common to every β-Gal positive cell observed in the reporter mouse in these studies [27]–[30].

Strong expression of the metabotropic glutamate receptor 1α (mGluR1α) is also a hallmark of a majority of UBCs. Consequently, anti-mGluR1α staining has been used previously to demonstrate expression of this type of interneuron in the granular layer of lobules X and IXb-c [19], [31], [32]. As shown in figure S2, interneurons labeled with anti-mGluR1α antibody in the wild type mouse have an expression pattern similar to that of those positive for β-Gal in the *Asic5^tm2a(KOMP)Wtsi^* mouse. Both are enriched in the granular layers of lobules X and IXb and IXc, but not IXa. (The modest labeling of Purkinje cells by anti-β-Gal antibody shown in figure S2 is nonspecific as it was also observed in the cerebellum of wild type animals, as shown in figure S3, and as compared to labeling of UBCs which was specific to the reporter animal and not observed in wild type littermates.)

To further establish the identity of *Asic5* expressing cells, sagittal sections of the cerebellum of the *Asic5^tm2a(KOMP)Wtsi^* reporter mouse were co-labeled with anti-mGluR1α and anti-β-Gal antibodies. As shown in figure 4 and figure S4, anti-mGluR1α and β-Gal antibodies labeled the same interneuron with the prior antibody primarily labeling dendriole brushes as expected.

### *Asic5* is expressed chiefly in mGluR1α (+) UBCs

There are at least two major subtypes of UBCs. Those that richly express mGluR1α termed mGluR1α (+) and those that express calretinin termed CR (+) [19], [32]. The prior represents the majority, approximately 2/3, of the UBCs in the vestibulocerebellum and the latter about 1/3. Interestingly, mGluR1α (+) but not CR (+) UBCs are expressed throughout lobules X and IXb-c [19]. In comparison, CR (+) UBCs are expressed only in lobule X and the anterior portion of lobule IXc. Thus, the expression pattern of mGluR1α (+) UBCs best corresponds with those expressing *Asic5* as assessed in the reporter mouse. To further explore this possibility, we co-labeled sagittal sections from the reporter mouse with anti-calretinin and anti-β-Gal antibodies. As is clear in the representative fluorescence micrograph shown in figure S5, anti-CR and anti-β-Gal antibodies label distinct populations of UBCs. Indeed, no cell was found to be co-stained by both antibodies in these experiments.

**Figure 5:**
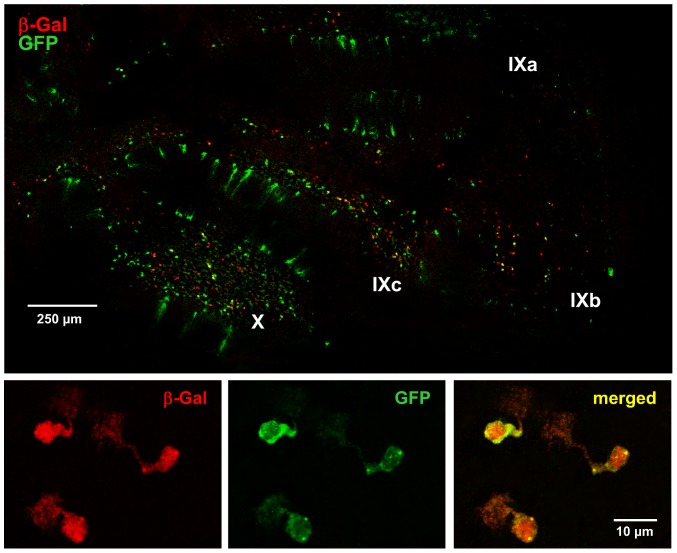
*Asic5* is expressed in GFP-positive vestibulocerebellar interneurons in the Tg(Grp-EGFP)DV197Gsat mouse. A representative fluorescence micrograph of a typical midsagittal section through the vermis of the cerebellum of a mouse that contains at least one copy of the Tg(Grp-EGFP) transgene and that is also homozygous for the *Asic5^tm2a(KOMP)Wtsi^* reporter allele. (This experiment was repeated with similar results in more than four different mice.) This section was probed with anti-β-Gal (red) and anti-GFP (green) antibodies. A region of lobule IX that contains typical UBCs positive for both β-Gal (red; left) and GFP (green; middle) is shown at a magnified scale below. The merged image is shown to the right.

As shown previously, GFP expression in the Tg(Grp-EGFP)DV197Gsat mouse is chiefly in mGluR1α (+) UBCs [18], [19]. The current study took advantage of this. Figure 5 shows a representative midsagittal section through the vermis of the cerebellum stained with anti-GFP and anti-β-Gal antibodies from a mouse homozygous for the *Asic5^tm2a(KOMP)Wtsi^* reporter allele and that also had at least one copy of the Tg(Grp-EGFP) transgene. Similar to mGluR1α as shown above, GFP expression in these animals strongly overlapped with that of *Asic5*, as assessed by expression of β-Gal, with significant co-localization in interneurons in the granular layer of lobules X and IXb-c.

**Figure 6:**
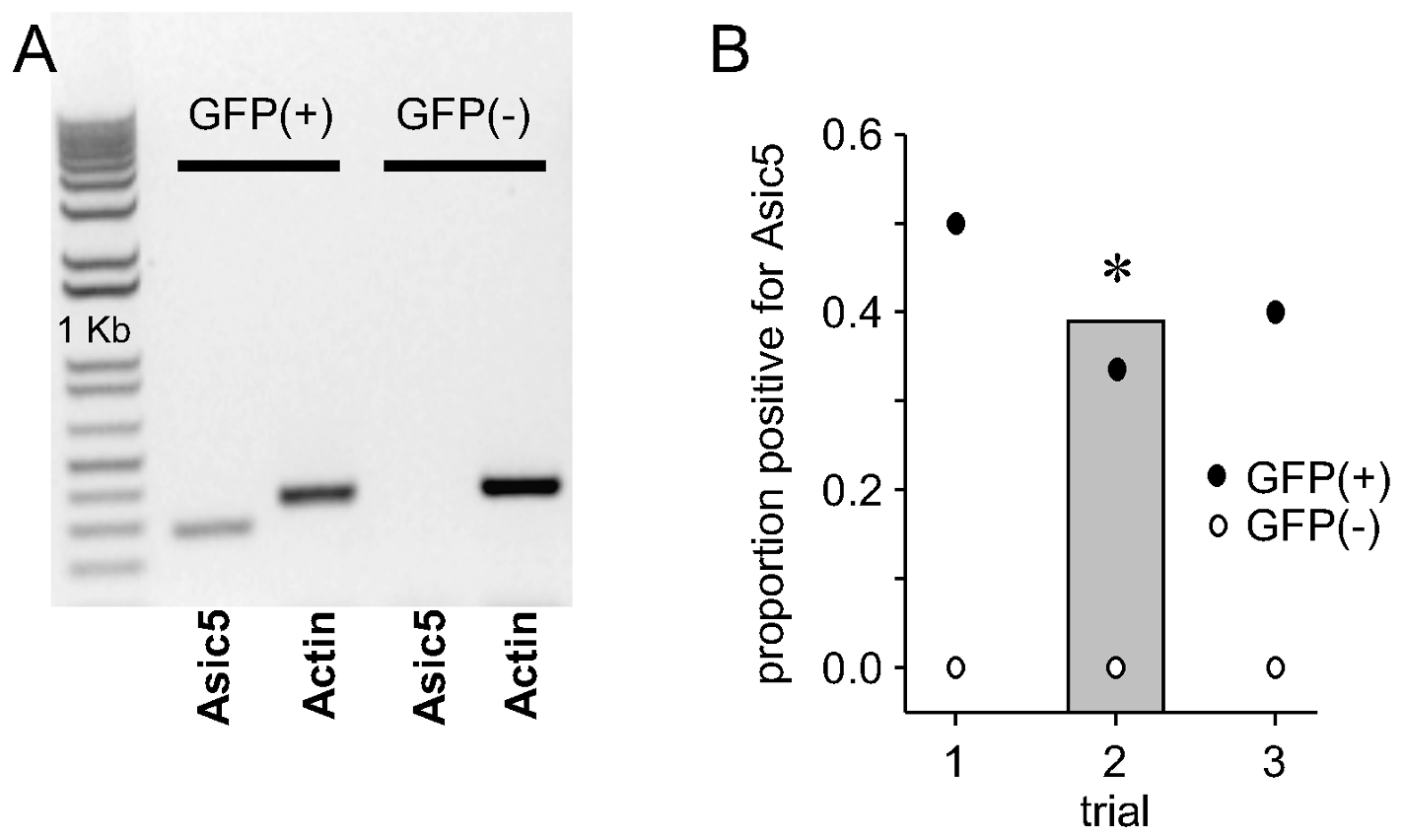
Asic5 mRNA is restrictively expressed in mGluR1α (+) UBCs. A. Inverted image of a representative gel containing typical single-cell RT-PCR products from representative GFP-positive and GFP-negative vestibulocerebellar neurons from the Tg(Grp-EGFP)DV197Gsat mouse. Each cell was probed for expression of Asic5 mRNA with actin serving as an internal reaction control. B. Graph showing the proportion of GFP-positive and GFP-negative cells that expressed Asic5 mRNA in single-cell RT-PCR experiments using neurons from the vestibulocerebellum of three different Tg(Grp-EGFP)DV197Gsat mice. In this figure, open and closed circles show the proportion of GFP-negative and positive cells, respectively, that also expressed Asic5 mRNA in these three trials. In each of these trials more than four interneurons were tested per animal. The gray bar in this figure shows the total proportion of GFP-positive vestibulocerebellar interneurons from the Tg(Grp-EGFP)DV197Gsat mouse that also expressed *Asic5*. ^*^Significantly larger proportion as compared to GFP-negative cells.

Single-cell RT-PCR findings from the Tg(Grp-EGFP)DV197Gsat mouse further support that a significant portion of mGluR1α (+) UBCs express Asic5. A representative observation and corresponding summary graph of such experiments are shown in figure 6. A significant proportion of GFP-positive UBCs in the Tg(Grp-EGFP)DV197Gsat mouse expressed Asic5 mRNA. In contrast, Asic5 mRNA was never observed in cerebellar neurons that lacked GFP in the transgenic animal.

### UBCs enriched in *Asic5* have electrical properties consistent with those positive for mGluR1α

The electrophysiological properties of granular cells (GC) and GFP-positive UBCs in the transgenic animal, which as shown above are enriched in *Asic5*, were determined next in vestibulocerebellar slices. As shown by the typical traces from representative current clamped GCs and GFP-positive UBCs, and corresponding summary graphs of resting membrane potential, input resistance and capacitance in figure S6, GCs and GFP-positive UBCs were distinguishable by their respective electrical properties. As compared to GCs, the input resistance and capacitance of GFP-positive UBCs in the Tg(Grp-EGFP)DV197Gsat mouse were lesser and greater in magnitude, respectively. The resting membrane potential of GFP-positive UBCs was also more depolarized compared to GCs. These findings are consistent with similar electrophysiological results published previously showing that the majority of GFP-positive interneurons in the the Tg(Grp-EGFP)DV197Gsat mouse are mGluR1α (+) UBCs [19], [25], [33].

As compared to GCs and CR (+) UBCs which intrinsically and regularly fire action potentials, mGluR1α (+) UBCs fire little at rest and show a bursting phenotype when stimulated [19], [25], [33]. This was reaffirmed in the present study as shown in figure S7. This provides further evidence that the population of UBCs enriched in *Asic5*, as followed by GFP expression in the transgenic animal, are those that express mGlurR1α. Current clamped GCs and GFP-positive UBCs in vestibulocerebellar slices showed hallmark continuous and bursting firing patterns, respectively, in response to injection of a depolarizing supra-threshold current. In addition to differences in characteristic firing patterns, the first and second action potentials in evoked trains of action potentials from GCs and GFP-positive UBCs had distinct properties. Action potentials in GFP-positive UBCs as compared to GCs had shorter inter-event intervals, were broader and had markedly smaller fast after hyperpolarizing potentials. In addition, whereas the 1^st^ action potential in GFP-positive UBCs reached a significantly greater peak voltage compared to that in GCs, the next action potential peaked at a significantly lower voltage compared to the corresponding action potential in GCs.

## Discussion

The current findings demonstrate that *Asic5* is restrictively expressed in the brain in a subset of specialized interneurons that are localized to the granular layer of the vestibulocerebellum. The cerebellar interneurons which express *Asic5* are UBCs with this channel chiefly expressed in UBCs that also express mGluR1α. This restrictive expression pattern has important implications for the cellular and physiological functions of this channel.

The current study makes several observations in support of Asic5 being expressed in a restrictive manner in mGluR1α (+) UBCs of the vestibulocerebellum. The *Asic5* reporter mouse shows little expression in the brain outside of the cerebellum but rich expression in this region. This expression profile agrees with that reported for *Asic5* in the Allen Mouse Brain Atlas (Website: 2012 Allen Institute for Brain Science. Allen Mouse Brain Atlas [Internet]. Available from: http://mouse.brain-map.org/) [34]. Though, it is easily missed in the latter resource because of the restrictive and punctuate nature of *Asic5* expression in the brain and because the transcript for this channel, similar to that for most other ion channels, likely has a low copy number. In the cerebellum, expression is localized to the vestibulocerebellum. In the vestibulocerebellum expression is in interneurons in the granular layer of lobules X and IXb and IXc but not IXa. This is the region of the cerebellum where UBCs are found [19], [28], [31]. UBCs are sparse in other cerebellar folia. Like mGluR1α (+) UBCs [19], β-Gal positive interneurons in the *Asic5^tm2a(KOMP)Wtsi^* reporter mouse are also richly expressed in lobules X and IXc and throughout IXb. In comparison, CR (+) UBCs are enriched in lobule X and the anterior portion of IXc but sparse in the posterior portion of IXc and the entirety of IXb [19], [31].

The cellular morphology of β-Gal expressing cells in the granular layer of the vestibulocerebellum of the *Asic5^tm2a(KOMP)Wtsi^* reporter mouse is also consistent with them being UBCs. They have the hallmark single short but broad dendrite of UBCs that terminates in the characteristic expansive dendriole brush [27], [29], [30], [35].

The immunohistochemical properties of β-Gal expressing interneurons in the reporter mouse in addition also are consistent with them being mGluR1α (+) UBCs. A significant proportion of the β-Gal expressing interneurons in the reporter mouse also express mGluR1α. In contrast, no β-Gal positive cell was positive for CR. Moreover, there is notable overlap between the population of interneurons that express β-Gal in the granular layer of the vestibulocerebellum in the reporter mouse and those that express GFP when the Tg(Grp-EGFP) transgene is introduced into this animal. GFP-positive cells in the Tg(Grp-EGFP)DV197Gsat mouse are recognized to be primarily mGluR1α (+) UBCs [19].

Independent evidence consistent with Asic5 being restrictively expressed in mGluR1α (+) UBCs comes from the observation that a significant portion of the GFP-positive but none of the GFP-negative cerebellar neurons tested in the Tg(Grp-EGFP)DV197Gsat mouse expressed Asic5 mRNA.

The final piece of evidence in these studies consistent with Asic5 being enriched in mGluR1α (+) UBCs is that the GFP-positive cells, many of which also express β-Gal, in the *Asic5* reporter/Tg(Grp-EGFP) transgenic mouse had electrical properties consistent with that reported previously for mGluR1α (+) UBCs [19], [25], [26], [29].

Considering its restrictive expression in the brain in mGluR1α (+) UBCs, an important question is what is the function of Asic5 in these interneurons, and why do these cells in particular express this channel when other similar types of interneurons, for instance CR (+) UBCs in the vestibulocerebellum and granular cells in the wider cerebellum, do not appear to express this channel? Of the mammalian Asic and ENaC proteins, Asic5 is the most closely related to ancestral Deg/ENaC progenitors, including the neuropeptide-gated FaNaC and HyNaC channels expressed in mollusks and hydra, respectively [5], [13], [14]. Indeed, Asic5 shares approximately as much sequence identity with these primordial Deg/ENaC proteins as it does with other mammalian Asic proteins [13]. Ancestral Deg/ENaC proteins expressed in simpler animals in which nervous systems first developed, such as the hydra, function as post-synaptic ionotropic receptors for neuropeptides, as typified by RFamides, involved in fast synaptic transmission [1], [36]. Perhaps Asic5 is an ortholog of these ancestral channels that serves a similar function in mammals specifically in one of the oldest parts of the brain, the archicerebellum, which is another name for the vestibulocerebellum.

Irrespective of the specific function served by Asic5 in mGluR1α (+) UBCs, the current findings are consistent with Asic5 being restrictively expressed in this subset of interneurons. This finding by its very nature argues that the function of Asic5 in these cells is critical and distinctive. Moreover, in consideration of the work previously done on Asic5 in liver epithelia [15], [16], the current findings demonstrate that this channel performs at least two very distinct functions depending on whether it is expressed in neurons or epithelial cells. In neurons it most likely serves a function akin to that of other Asics and in epithelial cells one similar to ENaC.

## Supporting Information

Figure S1 β-Gal expression outside of the cerebellum in the brain of the *Asic5^tm2a(KOMP)Wtsi^* mouse. Representative midsagittal section (200 µm) through the brain of an *Asic5^tm2a(KOMP)Wtsi^* mouse stained for β-galactosidase activity. Areas of the cortex and olfactory tubercle are shown at a magnified scale. Dashed black lines show the relative positions of these magnified regions in the whole brain - Arrows note staining. The white asterisk notes staining in the vestibulocerebellum similar to that shown at higher magnification in figures 2 & 3.
(PDF)

Figure S2 *Asic5* is restrictively expressed in unipolar brush cells. Fluorescence micrographs showing lobules IX and X in typical midsagittal sections of the cerebellum from *Asic5^tm2a(KOMP)Wtsi^* (A) and *Asic5* wild type (B) mice stained with anti-β-Gal (green; A) and anti-mGluR1α (red; B) antibodies, respectively. An area of lobule X from the reporter mouse that contains a representative β-Gal positive interneuron is shown at a magnified scale (boxed). The cell soma and hallmark dendriole brush for this UBC are noted. Modest labeling of Purkinje cells by the β-Gal antibody is nonspecific as it is also present in cerebellum from wild type animals (see figure S3).
(PDF)

Figure S3 Non-specific staining of Purkinje cells by the anti-β-Gal antibody. Representative fluorescence micrographs showing lobules IX and X in typical midsagittal sections of the cerebellum from *Asic5* wild type mice stained with anti-β-Gal (green) antibody.
(PDF)

Figure S4 mGluR1α (+) UBCs express β-Gal in the *Asic5^tm2a(KOMP)Wtsi^* mouse. A fluorescence micrograph showing a close-up view of a typical mGluR1α (+) UBC in lobule X of the *Asic5^tm2a(KOMP)Wtsi^* reporter mouse that also expressed β-Gal. Anti-mGluR1α and β-Gal staining are red and green, respectively. The cell soma and hallmark dendriole brush of this representative UBC are noted.
(PDF)

Figure S5 CR (+) UBCs do not express β-Gal in the *Asic5^tm2a(KOMP)Wtsi^* mouse. A representative fluorescence micrograph showing lobules IX and X in a typical midsagittal section of the cerebellum from an *Asic5^tm2a(KOMP)Wtsi^* reporter mouse stained with anti-CR (red) and anti-β-Gal (green) antibodies. Areas from lobule X containing CR (+) and β-Gal-positive UBCs are shown at a magnified scale (boxed). The cell soma and hallmark dendriole brushes of these representative types of UBCs are noted.
(PDF)

Figure S6 Electrical properties of GFP-positive vestibulocerebellar interneurons in the Tg(Grp-EGFP)DV197Gsat mouse. A. Representative traces from typical GCs (middle) and GFP-positive UBCs (bottom) in vestibulocerebellar slices from a wild type and Tg(Grp-EGFP)DV197Gsat mouse. These interneurons were current-clamped at rest and then subjected to a hyperpolarizing (−20 pA) current injection (waveform shown at top). Inset shows a clamped UBC that was back-filled with lucifer yellow to allow post-hoc analysis of morphology. The dendriole brush and soma of this UBC are noted. B. Summary graphs of the mean resting membrane potential (top), input resistance (middle) and capacitance (bottom) of GCs and GFP-positive UBCs in vestibulocerebellar slices. Data collected from experiments identical to that in S6A. Also shown in these graphs are the individual data points collected for each cell type. Individual GCs and UBCs were recorded from both wild type and Tg(Grp-EGFP)DV197Gsat mice with the bulk of the data points for the former coming from the wild type animal and the latter the transgenic animal.
(PDF)

Figure S7 GFP-positive vestibulocerebellar interneurons of the Tg(Grp-EGFP)DV197Gsat mouse have a distinctive bursting phenotype and action potential shape. A. Representative trains of action potentials in current-clamped GCs (top) and GFP-positive UBCs (bottom) in vestibulocerebellar slices evoked by a 30 pA supra-threshold current injection (current pulse shown at the top). B. Overlays of typical 1^st^ (top) and 2^nd^ (bottom) action potentials in GCs (gray) and UBCs (black) evoked by a supra-threshold current injection. C. Summary graphs (n>8) of inter-event interval, the action potential peak amplitude, 10% width and fast after hyperpolarization (*fAHP*) for the 1^st^ and 2^nd^ action potentials from GCs (gray) and UBCs (black). ^*^Significantly different.
(PDF)
