# Supplementary figures and images for: Restrictive Expression of Acid-Sensing Ion Channel 5 (Asic5) in Unipolar Brush Cells of the Vestibulocerebellum
Source: PLoS One. 2014 Mar 24;9(3):e91326. doi: 10.1371/journal.pone.0091326 (PMC3963869; doi:10.1371/journal.pone.0091326)

**Figure S1**

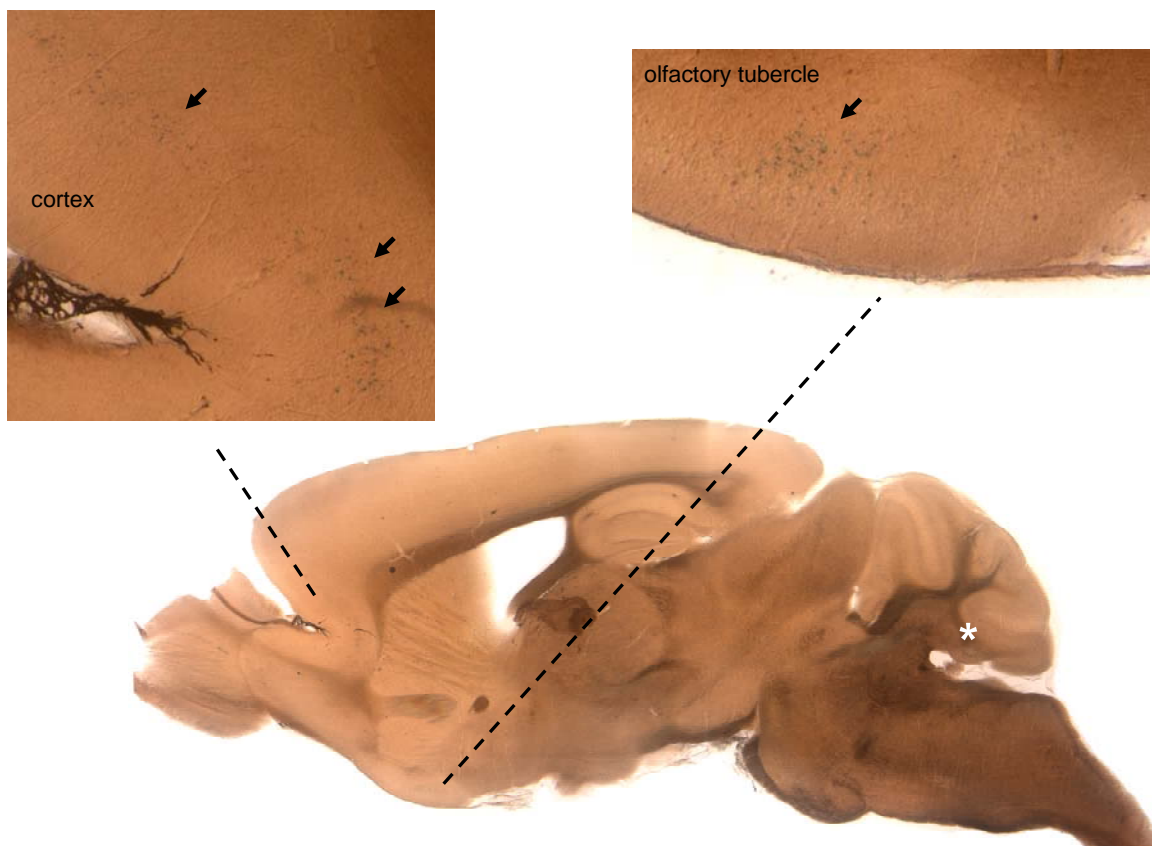

Supplement: Figure S1 — β-Gal expression outside of the cerebellum in the brain of the Asic5tm2a(KOMP)Wtsi mouse. Representative midsagittal section (200 µm) through the brain of an Asic5tm2a(KOMP)Wtsi mouse stained for β-galactosidase activity. Areas of the cortex and olfactory tubercle are shown at a magnified scale. Dashed black lines show the relative positions of these magnified regions in the whole brain - Arrows note staining. The white asterisk notes staining in the vestibulocerebellum similar to that shown at higher magnification in figures 2 & 3. (PDF) [file pone.0091326.s001.pdf]

Figure S2

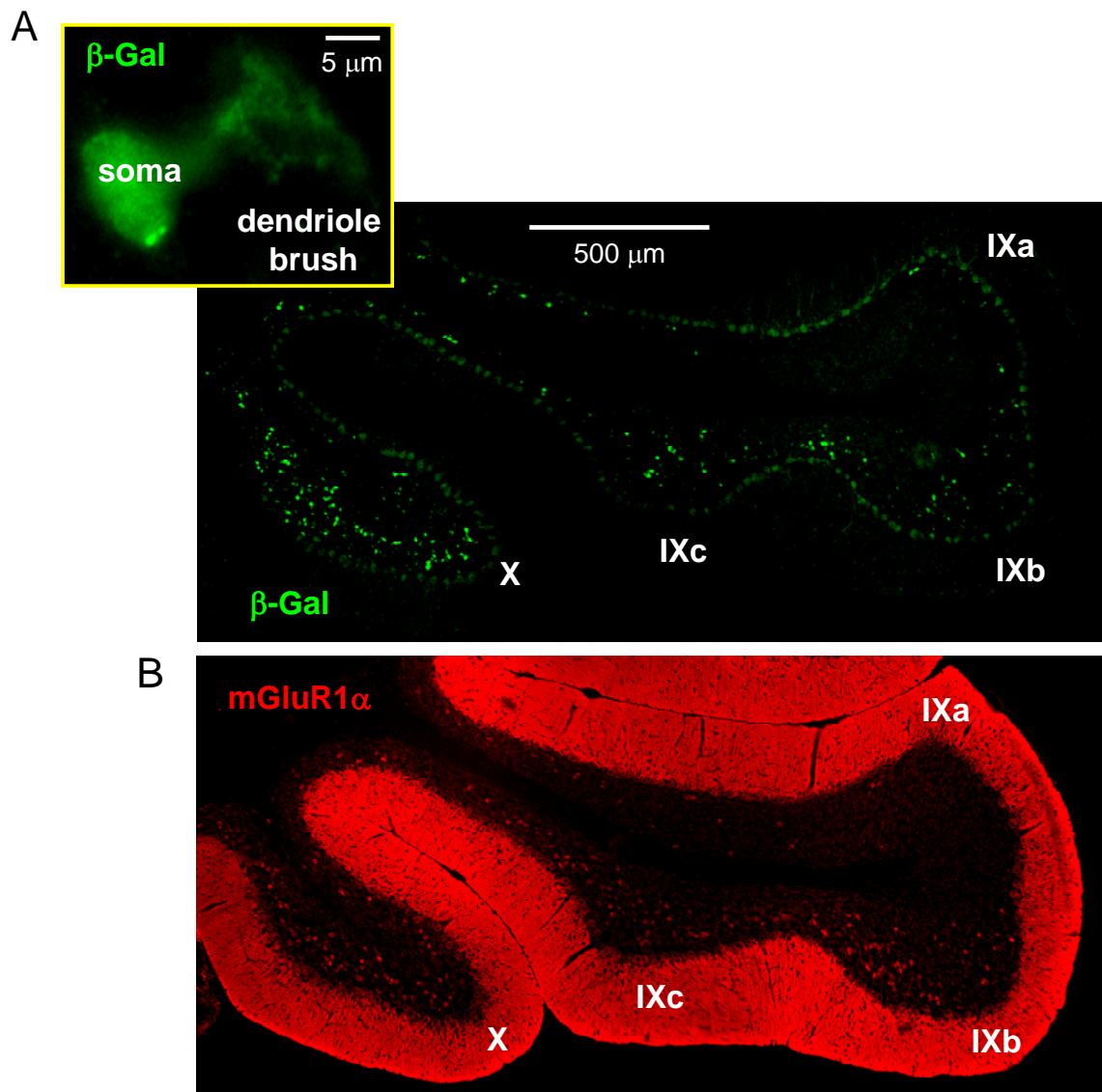

Supplement: Figure S2 — Asic5 is restrictively expressed in unipolar brush cells. Fluorescence micrographs showing lobules IX and X in typical midsagittal sections of the cerebellum from Asic5tm2a(KOMP)Wtsi (A) and Asic5 wild type (B) mice stained with anti-β-Gal (green; A) and anti-mGluR1α (red; B) antibodies, respectively. An area of lobule X from the reporter mouse that contains a representative β-Gal positive interneuron is shown at a magnified scale (boxed). The cell soma and hallmark dendriole brush for this UBC are noted. Modest labeling of Purkinje cells by the β-Gal antibody is nonspecific as it is also present in cerebellum from wild type animals (see figure S3). (PDF) [file pone.0091326.s002.pdf]

Figure S3

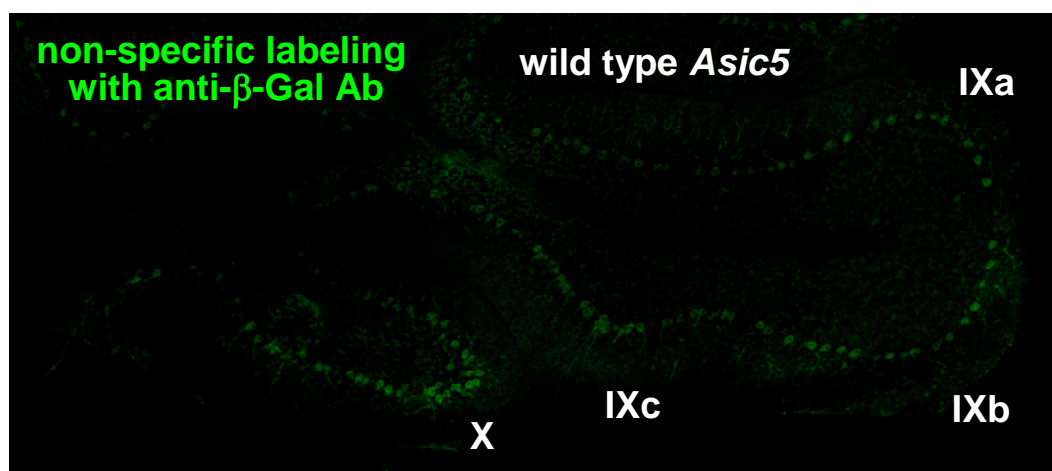

Supplement: Figure S3 — Non-specific staining of Purkinje cells by the anti-β-Gal antibody. Representative fluorescence micrographs showing lobules IX and X in typical midsagittal sections of the cerebellum from Asic5 wild type mice stained with anti-β-Gal (green) antibody. (PDF) [file pone.0091326.s003.pdf]

Figure S4

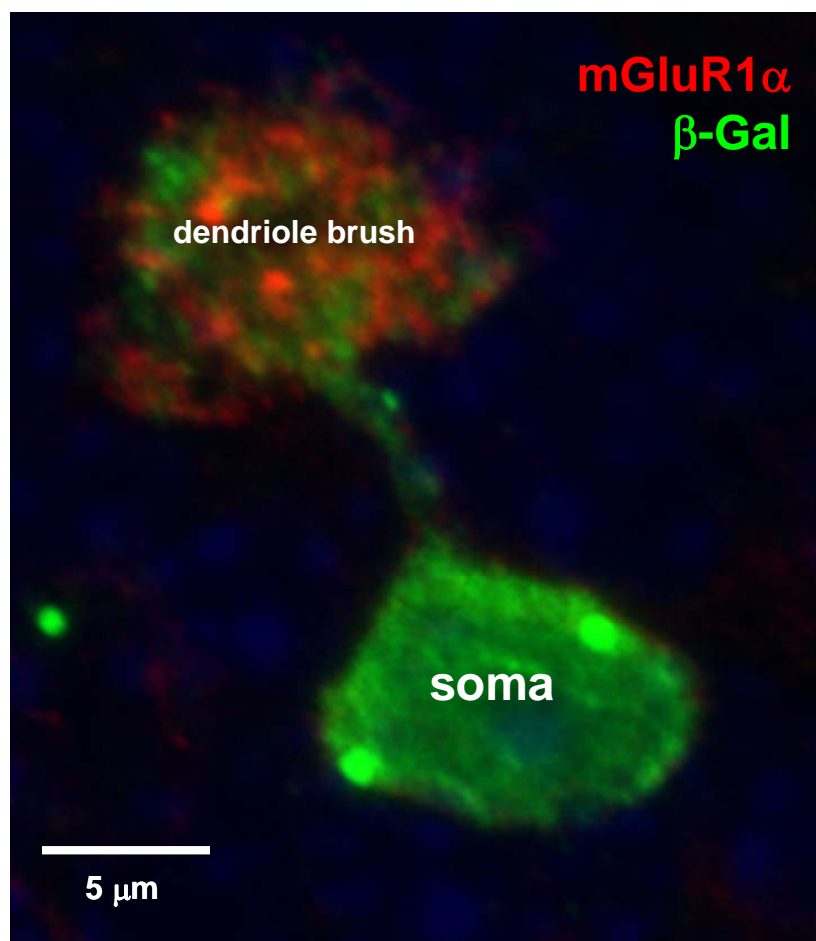

Supplement: Figure S4 — mGluR1α (+) UBCs express β-Gal in the Asic5tm2a(KOMP)Wtsi mouse. A fluorescence micrograph showing a close-up view of a typical mGluR1α (+) UBC in lobule X of the Asic5tm2a(KOMP)Wtsi reporter mouse that also expressed β-Gal. Anti-mGluR1α and β-Gal staining are red and green, respectively. The cell soma and hallmark dendriole brush of this representative UBC are noted. (PDF) [file pone.0091326.s004.pdf]

Figure S5

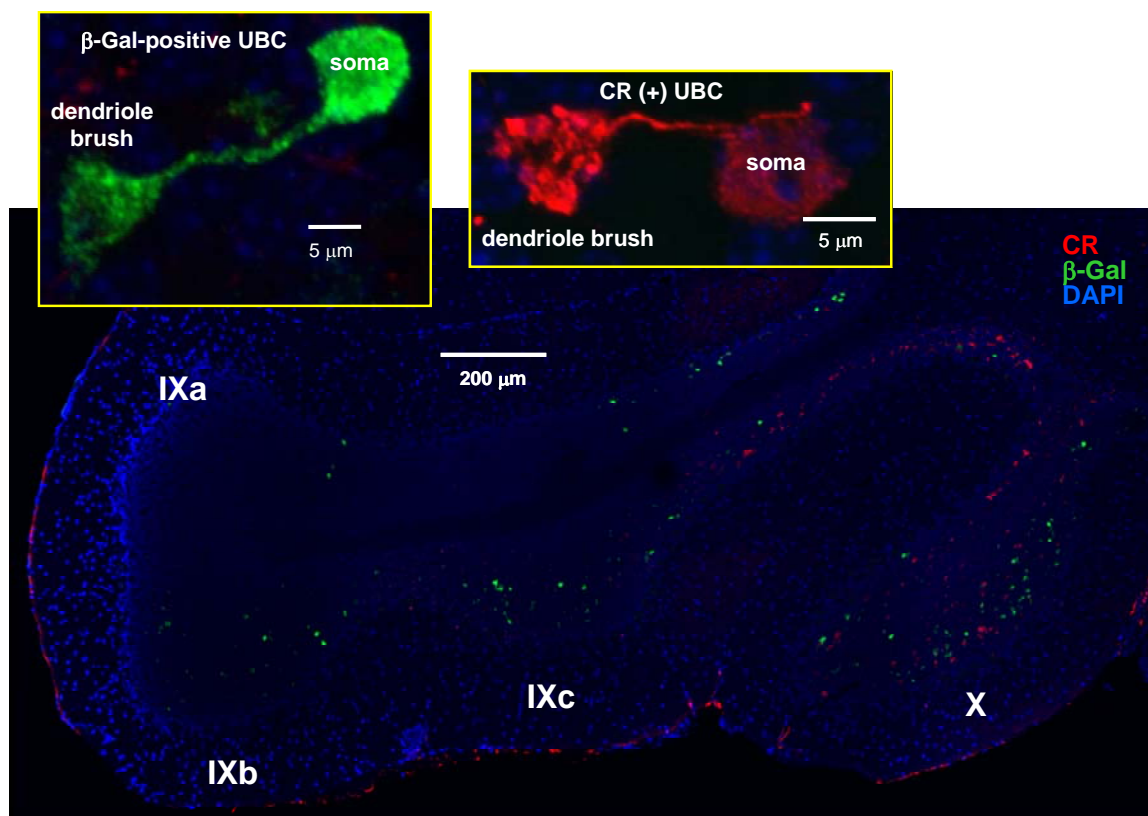

Supplement: Figure S5 — CR (+) UBCs do not express β-Gal in the Asic5tm2a(KOMP)Wtsi mouse. A representative fluorescence micrograph showing lobules IX and X in a typical midsagittal section of the cerebellum from an Asic5tm2a(KOMP)Wtsi reporter mouse stained with anti-CR (red) and anti-β-Gal (green) antibodies. Areas from lobule X containing CR (+) and β-Gal-positive UBCs are shown at a magnified scale (boxed). The cell soma and hallmark dendriole brushes of these representative types of UBCs are noted. (PDF) [file pone.0091326.s005.pdf]

**Figure S6**

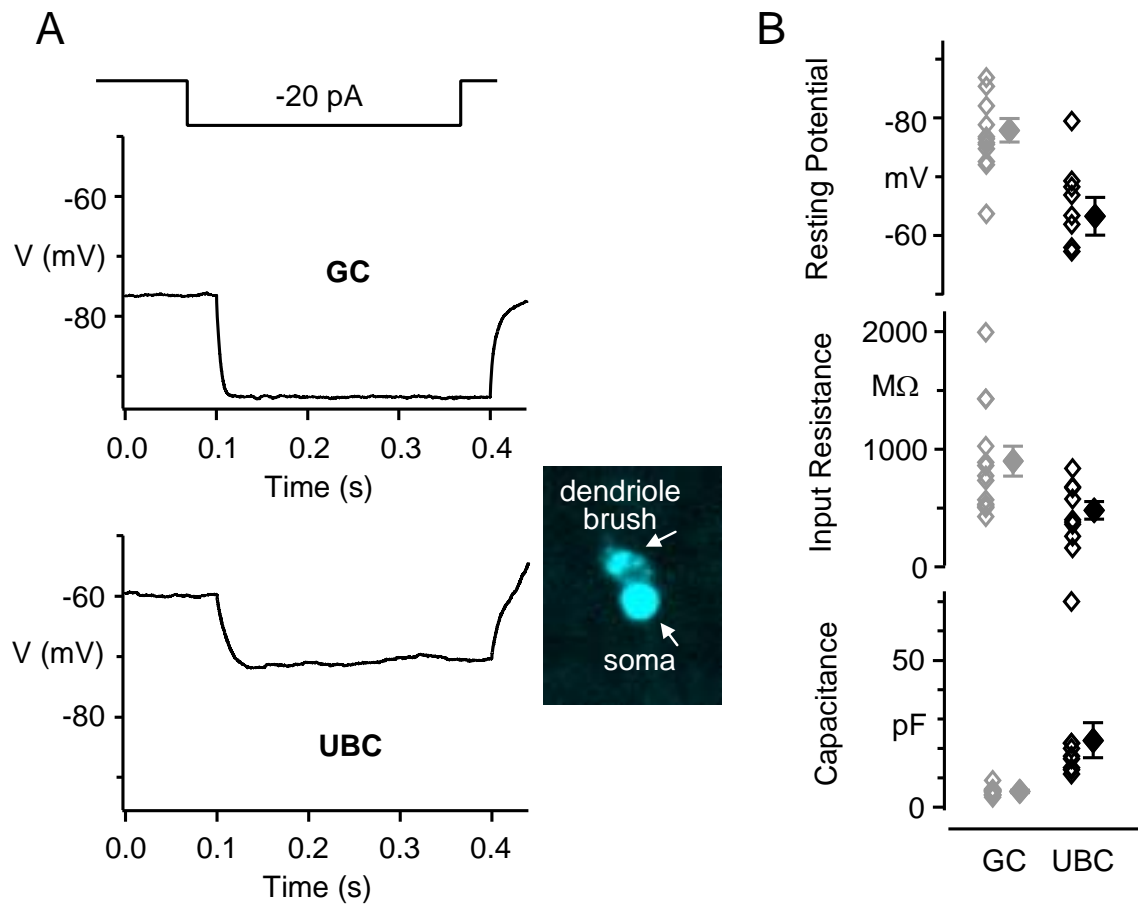

Supplement: Figure S6 — Electrical properties of GFP-positive vestibulocerebellar interneurons in the Tg(Grp-EGFP)DV197Gsat mouse. A. Representative traces from typical GCs (middle) and GFP-positive UBCs (bottom) in vestibulocerebellar slices from a wild type and Tg(Grp-EGFP)DV197Gsat mouse. These interneurons were current-clamped at rest and then subjected to a hyperpolarizing (−20 pA) current injection (waveform shown at top). Inset shows a clamped UBC that was back-filled with lucifer yellow to allow post-hoc analysis of morphology. The dendriole brush and soma of this UBC are noted. B. Summary graphs of the mean resting membrane potential (top), input resistance (middle) and capacitance (bottom) of GCs and GFP-positive UBCs in vestibulocerebellar slices. Data collected from experiments identical to that in S6A. Also shown in these graphs are the individual data points collected for each cell type. Individual GCs and UBCs were recorded from both wild type and Tg(Grp-EGFP)DV197Gsat mice with the bulk of the data points for the former coming from the wild type animal and the latter the transgenic animal. (PDF) [file pone.0091326.s006.pdf]

**Figure S7**

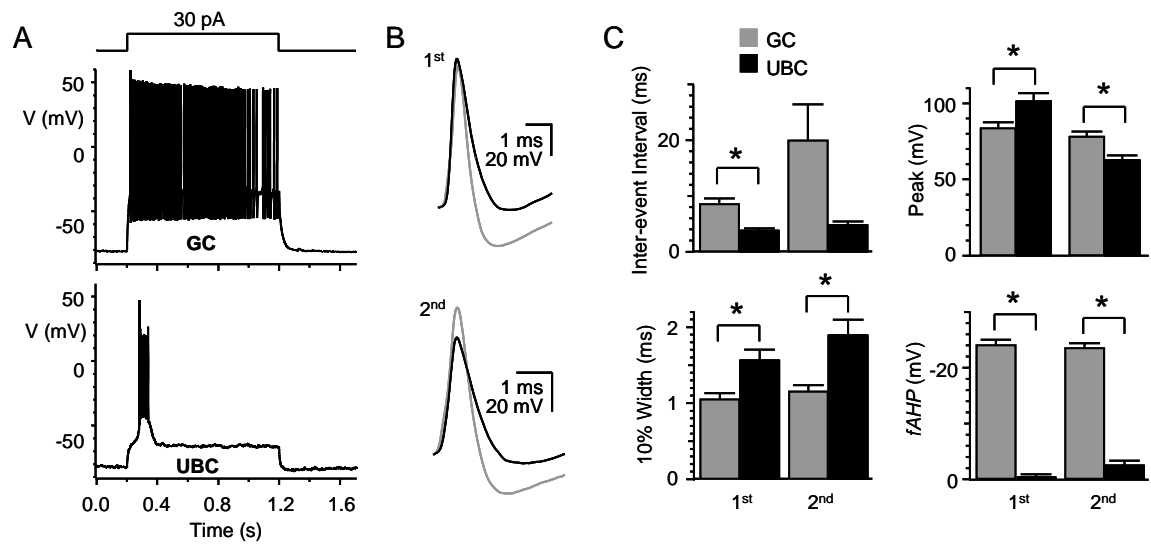

Supplement: Figure S7 — GFP-positive vestibulocerebellar interneurons of the Tg(Grp-EGFP)DV197Gsat mouse have a distinctive bursting phenotype and action potential shape. A. Representative trains of action potentials in current-clamped GCs (top) and GFP-positive UBCs (bottom) in vestibulocerebellar slices evoked by a 30 pA supra-threshold current injection (current pulse shown at the top). B. Overlays of typical 1st (top) and 2nd (bottom) action potentials in GCs (gray) and UBCs (black) evoked by a supra-threshold current injection. C. Summary graphs (n>8) of inter-event interval, the action potential peak amplitude, 10% width and fast after hyperpolarization (fAHP) for the 1st and 2nd action potentials from GCs (gray) and UBCs (black). *Significantly different. (PDF) [file pone.0091326.s007.pdf]
